# Empowering Strategies for Lifestyle Interventions, Diet Modifications, and Environmental Practices for Uterine Fibroid Prevention; Unveiling the LIFE UP Awareness

**DOI:** 10.3390/nu16060807

**Published:** 2024-03-12

**Authors:** Somayeh Vafaei, Samar Alkhrait, Qiwei Yang, Mohamed Ali, Ayman Al-Hendy

**Affiliations:** Department of Obstetrics and Gynecology, University of Chicago, Chicago, IL 60637, USA; somayeh.vafaei@gmail.com (S.V.); skhrait@bsd.uchicago.edu (S.A.); yangq@bsd.uchicago.edu (Q.Y.)

**Keywords:** UFs, lifestyle, nutrition, environmental, prevention

## Abstract

Uterine fibroids (UFs) are the most common prevalent benign tumor among women of reproductive age, disproportionately affecting women of color. This paper introduces an innovative management strategy for UFs, emphasizing the curbing of disease prevention and progression. Traditionally, medical intervention is deferred until advanced stages, necessitating invasive surgeries such as hysterectomy or myomectomy, leading to high recurrence rates and increased healthcare costs. The strategy, outlined in this review, emphasizes UF disease management and is named LIFE UP awareness—standing for Lifestyle Interventions, Food Modifications, and Environmental Practices for UF Prevention. These cost-effective, safe, and accessible measures hold the potential to prevent UFs, improve overall reproductive health, reduce the need for invasive procedures, and generate substantial cost savings for both individuals and healthcare systems. This review underscores the importance of a proactive UF management method, paving the way for future research and policy initiatives in this domain.

## 1. Introduction

Uterine fibroids (UFs), also known as leiomyoma, are a predominant global health concern impacting numerous women in their reproductive years. Historically, UFs were just monitored until symptoms like heavy menstrual bleeding (HMB), anemia, painful periods, and pelvic pain necessitated surgical intervention. In recognizing the limitations of this watchful waiting approach, there’s a growing need for comprehensive prevention and management strategies [1].

Research is shedding light on the complex factors contributing to UFs, including genetic, environment, and lifestyle factors, paving the way for innovative prevention methods. This article delves into prevention methods, spotlighting the LIFE UP awareness (Lifestyle Interventions, Food Modifications, and Environmental Practices for UF Prevention). The goal is to empower women and healthcare professionals with practical insights to improve the quality of life (QOL) for those dealing with UFs. Known risk factors for UFs include age, race, BMI, pregnancy history, hypertension, vitamin D levels, hormonal imbalances, and exposure to endocrine-disrupting chemicals (EDCs). Genetic factors and lifestyle choices, such as diet and stress levels, also play a role. Recent research suggests that early exposure to these chemicals may reprogram myometrial stem cells, contributing to UF development [2,3].

Currently, there is no validated method for preemptively screening asymptomatic women for UF risk. Shear wave elastography (SWE) has been proposed as a potential screening tool capable of detecting subtle changes in myometrial tissue elasticity that may indicate a higher risk of UFs. Additionally, researchers are exploring urinary inflammatory biomarkers, alone or in combination with SWE, to identify presymptomatic women at risk [4,5]. Armed with this knowledge, primary preventive strategies are being developed to reduce UF prevalence. These strategies are safe and fertility friendly and aim to identify at-risk individuals early, enabling healthcare providers to offer targeted interventions, lifestyle adjustments, and personalized counseling to reduce UF development [6].

The diagnosis and treatment of UFs employ a range of techniques, from pelvic examinations and imaging techniques like ultrasonography, MRI, and CT scans [7]. Treatment options range from non-hormonal and hormonal medical awareness to surgical procedures, depending on the patient’s symptoms and goals. The costs associated with UFs in the United States are substantial, especially when complicated by HMB [8].

Addressing the recurrence of UFs after myomectomy is a substantial concern in the realm of secondary prevention. Myomectomy, while selectively removing clinically significant UFs and preserving the uterus, presents a risk of symptom resurgence and the potential need for additional procedures. Despite the high recurrence rates, established guidelines for preventing symptom recurrence or inhibiting new UFs growth post myomectomy are currently lacking. Our team explored a previous approach known as the ESCAPE (Evidence-Based Approach for Secondary Prevention) of UF management. Utilizing options such as vitamin D (4000 IU/day), EGCG (800 mg/day), and EDC-free products is deemed safe, efficacious, and economically viable for extended use by women who have undergone myomectomy, aiming to prevent UF recurrence. Our team is actively pursuing the application of the ESCAPE approach by preparing a clinical trial named ERADICATE-UF. The existing approach of watchful observation for early disease is recognized as a significant missed opportunity in the literature [9,10].

In conclusion, this article emphasizes the importance of holistic prevention and management strategies for UFs, spanning lifestyle modifications and targeted interventions. The challenge of preventing UF recurrence post myomectomy is also highlighted, underscoring the need for further research in this area.

## 2. Lifestyle Interventions

### 2.1. Dos (What Should Be Adopted)

#### 2.1.1. Boost Digestive Tract, Liver, and Kidney Functions

Consistent bowel movements coupled with a healthy gut flow and optimal enzyme function play a vital role in breaking down toxins, including phthalates, and facilitating their elimination from the body. The usage of specific herbs, under the guidance of an expert, such as milk thistle, green tea, and dandelion root, may provide potential benefits in safeguarding the liver against UF-related damage [11].

Milk thistle (*Silybum marianum*) is a well-known herb acknowledged for its hepatoprotective properties. It contains a compound called silymarin, known to possess antioxidant and anti-inflammatory effects. These properties can aid in shielding liver cells from harm and promoting their regeneration [12]. *Milk thistle*, along with green tea and dandelion root (*Taraxacum officinale*), has been utilized to support liver health and exhibit diuretic properties. It is believed to enhance bile flow, facilitating the digestion and elimination of toxins from the liver. Other herbs like *Uva ursi*, celery root, and parsley primarily assist kidney function and promote detoxification [13].

#### 2.1.2. Reduce the Burden of Chronic Lifestyle Diseases (CLDs)

The use of hormonal contraceptives such as birth control pills or intrauterine devices (IUDs) has been shown to reduce the risk of UFs. Women should consult with their healthcare providers to determine which contraceptive method is best for them [14]. Chronic lifestyle diseases (CLDs) exhibit common modifiable risk factors that contribute to their long-term development. Recognizing the socio-behavioral predictors can greatly assist in the establishment of impactful community-based programs focused on prevention, intervention, and treatment [15]. CLDs, such as cardiovascular diseases, diabetes, and certain types of cancer, often share similar underlying causes. Factors like an unhealthy diet, physical inactivity, tobacco use, excessive alcohol consumption, and stress contribute to the development and progression of these diseases over time [16,17]. In targeting these modifiable risk factors, it is possible to reduce the burden of CLDs in communities. Understanding the socio-behavioral predictors associated with CLDs is crucial for designing effective prevention, intervention, and treatment programs [18]. Socioeconomic factors, cultural influences, access to healthcare, education levels, and individual behaviors all play significant roles in the development and management of CLDs [19]. By taking these factors into account, community-based programs can be tailored to address the specific needs and challenges of the population. Prevention efforts should focus on promoting healthy behaviors and lifestyle choices. This includes initiatives to improve access to nutritious foods, promote physical activity, reduce tobacco and alcohol use, and enhance stress management skills. Community-wide awareness campaigns and educational programs can empower individuals to make informed decisions and adopt healthier habits [20]. Interventions should also target high-risk populations, providing support and resources to mitigate the impact of modifiable risk factors [21]. This may involve health screenings, counseling services, and access to affordable healthcare options. In addressing these factors at an individual and community level, the burden of CLDs can be reduced, and overall well-being can be improved. In summary, recognizing the shared modifiable risk factors and socio-behavioral predictors of chronic lifestyle diseases is essential for developing effective community-based programs. In addressing these factors through prevention, intervention, and treatment strategies, it is possible to make significant progress in reducing the prevalence and impact of CLDs on individuals and communities [22,23].

### 2.2. Don’ts (What Should Be Avoided)

#### 2.2.1. Minimize/Avoid Stress

The concept of “stress” permeates our daily lives, which presents both advantages and challenges for stress researchers. Numerous observational studies have demonstrated the detrimental impact of stress on health. A meta-analysis conducted by Qin H et al. revealed that chronic psychological stress may elevate the risk of UFs by 24% (OR, 1.24, 95% CI [1.15, 1.34]) [24]. Interestingly, they found a statistically significant association between chronic psychological stress and the risk of UFs, particularly among non-Hispanic Blacks [17]. The influence of chronic psychological stress extends to the activities of the Hypothalamic–Pituitary–Adrenal (HPA) and Hypothalamic–Pituitary–Gonadal (HPG) axes, both of which can contribute to the development or progression of UFs by affecting estrogen or progesterone levels and inducing reproductive dysfunction [25]. Furthermore, norepinephrine has the potential to promote the synthesis of pro-inflammatory cytokines by upregulating the activity of Nuclear Factor Kappa B (*NF-κB*) through key inflammation- or proliferation-related pathways such as those of *JNKs* and *p38-MAPKs*. This cascade of events ultimately leads to the onset of UFs. Moreover, chronic psychological stress has been associated with increased levels in multiple circulating inflammatory markers, including *IL-6*, *IL-1β*, and *IL-10* [26]. Therefore, the reported link between chronic psychological stress and the risk of UFs appears to have a strong racial/ethnic component, which may help explain the observed disparities in UFs among different racial and ethnic groups.

#### 2.2.2. Combat Sedentariness

The hypothesis that a high BMI contributes to an increased risk of UFs was supported by our study, specifically indicating a higher risk in premenopausal women [27,28]. Baird et al. found that women who engaged in at least four hours of vigorous physical activity per week were more likely to experience a decreased risk of UFs [29]. The relatively low levels of recreational exercise among women in China, compared to other countries, may explain why a similar protective effect was not observed between non-occupational activity and UFs [30]. BMI was considered a risk factor for UFs solely in premenopausal women. Prospective studies have consistently shown a significant association between an increased BMI and the risk of UFs. Obesity is believed to contribute to UF development primarily by elevating endogenous hormone levels, reducing serum hormone-binding globulin, altering estrogen metabolism during premenopausal conditions, and affecting the signaling controls of myometrial cells, such as insulin receptors, insulin-like growth factors, and peroxisome proliferator-activated receptors [31,32]. A prospective study has also reported that the positive effect of BMI on UFs seems to be limited to premenopausal and perimenopausal women, rather than postmenopausal women. It is plausible that an increased BMI in postmenopausal women may be associated with a decrease not only in estrogen levels, but also in UF growth factors such as *IGF-1* [33]. Obesity is associated with an increased risk of UFs. Maintaining a healthy body weight through regular physical activity and a balanced diet can help reduce this risk [34,35].

#### 2.2.3. Limit Alcohol Consumption

Limiting alcohol consumption is recommended for women in order to reduce the risk of UFs. Studies have consistently shown an association between alcohol intake and an increased risk of developing UFs. It is advisable for women to restrict their alcohol consumption to no more than one drink per day. Excessive alcohol consumption has been linked to various health risks, and UFs are among the conditions affected by alcohol intake [36,37].

Several studies have provided evidence of a positive association between alcohol consumption and the risk of UFs. The exact mechanism behind this relationship is not fully understood, but it is believed that alcohol may influence hormone levels and disrupt hormonal balance, which can contribute to the development of UFs. To minimize the risk of UFs, it is recommended that women limit their alcohol intake. It is important to note that individual factors and susceptibility to UFs may vary, and some women may be more sensitive to the effects of alcohol than others. Therefore, it is always advisable to consult with a healthcare professional for personalized recommendations based on one’s specific health situation. By adopting a responsible awareness to alcohol consumption and adhering to the recommended limits, women can take proactive steps to minimize their risk of developing UFs and maintain their reproductive health [38,39].

## 3. Optimal Dietary Choices to Consider (Food Modifications)

A poor diet can contribute to the development and progression of UFs. Research suggests that dietary factors play a role in the risk and growth of UFs, highlighting the importance of maintaining a healthy and balanced diet to reduce the likelihood of developing this condition. Several components of a poor diet have been associated with an increased risk of UFs [40,41]. These include a high intake of processed and refined foods, sugary beverages, unhealthy fats, and a low consumption of fruits [42], vegetables, and fiber-rich foods. Such dietary patterns may contribute to hormonal imbalances, inflammation, and oxidative stress, all of which can promote the growth of UFs. A diet high in processed and refined foods, including refined carbohydrates and added sugars, has been linked to an increased risk of UFs [43]. These foods have a high glycemic index, leading to rapid spikes in blood sugar levels and potentially influencing hormone levels in the body. Furthermore, consuming an excess of unhealthy fats, particularly saturated and trans fats, can contribute to inflammation and disrupt hormonal regulation. This may create an environment conducive to the development and growth of UFs. An insufficient intake of fruits, vegetables, and fiber-rich foods has also been associated with an elevated risk of UFs. These foods are rich in antioxidants, vitamins, and minerals that help combat oxidative stress and inflammation. Dietary fiber has been shown to have protective effects against UFs by aiding in hormonal balance and promoting regular bowel movements [44]. While the exact mechanisms through which a poor diet influences UFs are not fully understood, the cumulative effect of unhealthy dietary choices can contribute to hormonal disturbances, inflammation, and impaired immune function, all of which may contribute to the development and progression of UFs. To reduce the risk of UFs, adopting a healthy and balanced diet is crucial. This includes consuming a variety of whole foods, such as fruits, vegetables, whole grains, lean proteins, and healthy fats. Limiting the intake of processed and refined foods, sugary beverages, and unhealthy fats is also recommended. Incorporating dietary changes can be challenging, but it is important to prioritize long-term health benefits. Seeking guidance from healthcare professionals, nutritionists, or dieticians can provide valuable support and personalized recommendations for maintaining a healthy diet that may help reduce the risk of UFs.

### 3.1. Vegetables

An essential aspect of a diet aimed at preventing UFs is the inclusion of vegetables, particularly those that are rich in fiber. Vegetables provide valuable nutrients and contribute to the regulation of hormone levels, which is beneficial for UF prevention. Incorporating plant-based protein sources such as beans, lentils, whole grains, nuts, seeds, and lean meats can further support hormonal balance and facilitate regular bowel movements. By emphasizing these dietary elements, individuals can potentially reduce their risk of developing UFs and promote overall gynecological health [45,46].

#### 3.1.1. Leafy Green Vegetables

Leafy greens, including kale, spinach, collard greens, and Swiss chard, are excellent additions to a diet aimed at preventing UFs. These greens are not only packed with essential vitamins and minerals, but also contain high levels of antioxidants. These nutrients work together to combat inflammation in the body, which can contribute to the growth of UFs. By incorporating leafy greens into the diet, women can take advantage of their anti-inflammatory properties and potentially reduce the risk of UF development [47,48].

#### 3.1.2. Broccoli and Cauliflower

Cruciferous vegetables, such as broccoli and cauliflower, are particularly beneficial in the prevention of UFs. These vegetables contain natural compounds known as indoles, which have been found to help balance estrogen levels in the body [49,50]. By promoting estrogen balance, these indoles can potentially inhibit the growth of UFs [51,52].

#### 3.1.3. Carrots

Carrots are an excellent dietary source of beta-carotene, an antioxidant known for its anti-inflammatory properties and overall health benefits. Consuming carrots can contribute to reducing inflammation in the body. Moreover, carrots are rich in vitamin A, a vital nutrient that supports the maintenance of healthy tissues throughout the body [53].

#### 3.1.4. Sweet Potatoes

Sweet potatoes are a nutritious food choice, as they are not only delicious but also offer several health benefits. They are rich in dietary fiber, essential vitamins, and antioxidants that can play a role in regulating hormone levels and potentially prevent the growth of UFs. The fiber content in sweet potatoes supports healthy digestion and can contribute to hormonal balance. The vitamins and antioxidants present in sweet potatoes provide valuable support to the body’s overall health and well-being [54].

#### 3.1.5. Bell Peppers

Bell peppers are a vibrant and nutritious vegetable that offer numerous health benefits. They are particularly rich in vitamin C, which plays a crucial role in supporting a healthy immune system and promoting overall well-being. Consuming bell peppers can provide a significant boost to the immune system, helping the body fight off infections and illnesses. Moreover, bell peppers contain antioxidants, which are beneficial compounds that can help prevent the growth of UFs by combating oxidative stress and reducing inflammation [10,55].

### 3.2. Fruit

Scientific evidence suggests that a diet abundant in fruits can contribute to a reduced risk of UFs. Fruits are known to be rich in antioxidants and phytochemicals, which have anti-inflammatory properties. By incorporating fruits into the diet, women can potentially mitigate inflammation in the body and lower the risk of developing UFs. Furthermore, a study conducted by He et al., utilizing a validated self-administered questionnaire, found that higher intakes of vegetables and fruits, as well as engagement in occupational activities, were associated with protective effects against UFs. This suggests that consuming a variety of fruits and vegetables and maintaining an active occupational lifestyle may have a beneficial impact on UF prevention [27,46,55].

#### 3.2.1. Berries

Berries, including strawberries, raspberries, blackberries, and blueberries, are not only delicious, but also highly nutritious. These vibrant fruits are rich in antioxidants and anti-inflammatory compounds that offer a range of health benefits, including potentially preventing the growth of UFs. The antioxidants found in berries help combat oxidative stress and reduce inflammation in the body, which are factors that can contribute to UF development. By incorporating berries into the diet, women can harness the power of these antioxidant-rich fruits to promote overall health and potentially reduce the risk of UF growth [56]. In studies conducted on myometrial cells, it has been observed that various strawberry cultivars, such as Alba, Clery, Portola, Tecla, and Romina, exhibit beneficial effects. These cultivars have shown the ability to increase cellular viability and decrease the levels of reactive oxygen species (*ROS*) in myometrial cells. The findings suggest that the compounds present in strawberries may have antioxidant properties, contributing to improved cell health and reduced oxidative stress. These results highlight the potential of strawberries and their cultivars as valuable additions to promote cellular well-being in the context of myometrial health. Furthermore, studies have demonstrated that strawberries have the capacity to reduce the expressions *of fibronectin*, *collagen1A1*, and *Versican*, which are induced by activin A, in leiomyoma cells. Moreover, strawberries were found to reduce the expressions of activin A and plasminogen activator inhibitor-1 (*PAI-1*) mRNA in these cells. These findings suggest that strawberries possess anti-fibrotic properties, as they can attenuate the production of extracellular matrix (ECM) proteins and modulate activin A signaling pathways in leiomyoma cells [57].

#### 3.2.2. Citrus Fruits

Citrus fruits, including oranges, grapefruits, and lemons, are renowned for their refreshing taste and high vitamin C content. Vitamin C plays a vital role in bolstering the immune system and supporting overall health. Citrus fruits contain flavonoids, which are beneficial plant compounds known for their antioxidant properties. These flavonoids have the potential to help prevent the growth of UFs by reducing oxidative stress and inflammation in the body [58].

#### 3.2.3. Apples

Apples are not only a popular fruit, but also a valuable addition to a UF-preventive diet. They are a rich source of dietary fiber, which aids in maintaining healthy digestion and regulating hormone levels in the body. By promoting hormonal balance, apples may help prevent the growth of UFs. Furthermore, apples contain antioxidants, which play a crucial role in combating oxidative stress and reducing inflammation. These antioxidant properties contribute to overall health and may help protect against UF development [27,59].

#### 3.2.4. Pineapple

Pineapple is a tropical fruit known for its unique combination of flavors and health benefits. One notable component of pineapple is bromelain, an enzyme with anti-inflammatory properties. Bromelain has been associated with reducing inflammation in the body, which can be beneficial in managing various conditions, including UFs. Pineapples are rich in antioxidants, which play a vital role in protecting the body against oxidative stress and potentially preventing the growth of UFs [46,59].

#### 3.2.5. Kiwi

Kiwi is a fruit known for its vibrant green color and numerous health benefits. It is rich in vitamin C and antioxidants, which play essential roles in supporting a strong immune system and overall well-being. By incorporating kiwi into the diet, women can help boost the immune function. In addition, kiwi contains enzymes, such as actinidin, that have the ability to break down fibrin. Fibrin is a protein involved in blood clotting and can contribute to the growth of UFs. The presence of these enzymes in kiwi suggests that it may have a potential role in inhibiting or reducing the growth of UFs [60].

### 3.3. Fish

Fish, particularly fatty fish such as salmon, mackerel, and sardines, are rich in omega-3 fatty acids, possess anti-inflammatory properties and can offer potential advantages for individuals with UFs due to their nutritional composition. UFs are associated with chronic inflammation, and incorporating omega-3 fatty acids from fish into the diet may help mitigate inflammation in the body. Fish are a good source of high-quality protein, vitamins (such as vitamins D and B), and minerals (such as iron and magnesium), which are essential for overall health and well-being [61]. Omega-3 fatty acids, abundant in sources like fatty fish, flaxseed, chia seeds, walnuts, nuts, and seeds, possess anti-inflammatory properties that may have a beneficial impact on the risk of developing UFs. Several studies have indicated a potential link between a diet rich in omega-3 fatty acids and a reduced risk of UFs. The anti-inflammatory effects of omega-3 fatty acids may help modulate inflammation in the body, which is believed to play a role in UF development [62,63,64,65].

### 3.4. Poultry Products

Lean meats are a valuable addition to a balanced diet, and examples include skinless chicken, fish, and egg whites. The term “lean meats” encompasses various domesticated and captive bird species, bred for purposes such as egg production, meat production, or both. This includes commonly consumed poultry such as chicken, duck, turkey, geese, guinea fowl, and Japanese quail. These lean meat options offer high-quality protein, essential amino acids, and various nutrients while being lower in fat compared to other meat sources. Incorporating these lean meats into meals can provide a nutritious and flavorful component to support a healthy and well-rounded diet [66,67].

### 3.5. Vitamin D

Vitamin D, an essential fat-soluble vitamin, plays a pivotal role in maintaining various aspects of health, including calcium balance, bone health, and immune system modulation. There is growing interest in exploring its potential benefits in managing UFs due to the presence of vitamin D receptors in uterine tissue and associations between vitamin D deficiency and gynecological conditions like UFs [68]. Vitamin D supplementation, available in oral or injectable forms, is commonly recommended to address deficiencies and promote overall well-being. The active form of vitamin D, 1,25-dihydroxyvitamin D3, is crucial for regulating calcium and phosphate balance. It is synthesized in the skin upon exposure to sunlight and further converted in the liver and kidneys. Dietary sources of vitamin D include fatty fish, beef liver, egg yolks, and fortified dairy and grain products. Notably, vitamin D3 deficiency has been identified as a potential risk factor for UFs, contributing to lower serum vitamin D levels in affected women, especially among Black women who are more susceptible to deficiency. In UFs management, vitamin D has demonstrated anti-proliferative effects, inhibited UF growth and induced cell cycle arrest [69,70].

These effects are achieved through various molecular mechanisms, including the downregulation of *kinases*, *Bcl2*, and the suppression of the catechol-O-methyltransferase (COMT) expression. Animal studies have shown that a vitamin D-deficient diet can exacerbate UF-related gene expression, inflammation, and DNA damage. Furthermore, emerging evidence suggests that optimizing vitamin D levels may reduce the risk of UF recurrence by creating an environment less conducive to their growth. Vitamin D supplementation has also been linked to a decrease in fibroid size, offering a potential awareness to manage this condition. Clinical trials have reported significant reductions in UF size with long-term vitamin D supplementation, with an excellent safety profile and no reported adverse events [71,72]. The use of vitamin D (4000 IU/day) was introduced in our previous ESCAPE approach to manage UFs [10].

### 3.6. Decaffeinated Green Tea/EGCG

Green tea, rich in polyphenols like catechins, particularly epigallocatechin gallate (EGCG), has been studied for potential health benefits, including anti-inflammatory, antioxidant, and anti-cancer properties. Some studies suggest that drinking green tea on a full stomach may prevent uterine fibroids (UFs), but clinical data on its specific use for treating UFs are limited. Observational studies on the relationship between green tea consumption and UF incidence or progression have produced mixed results, exploring various mechanisms [73,74].

**Figure 1:**
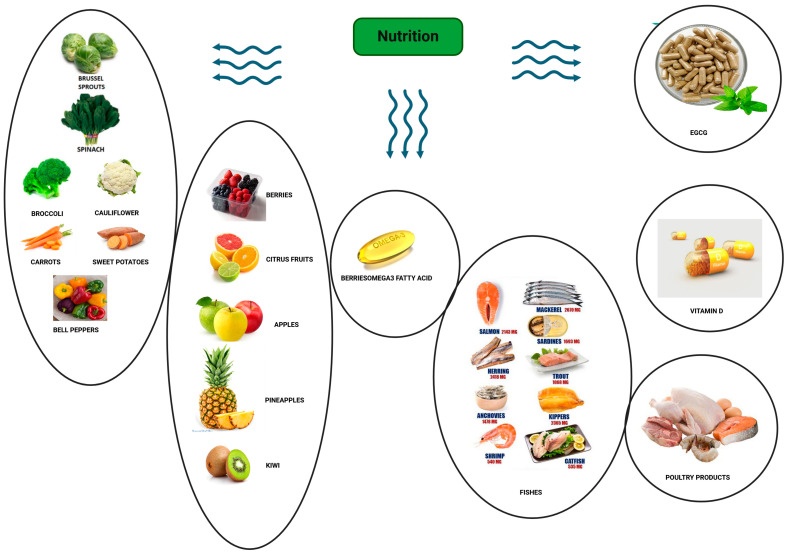
Nutrition in uterine fibroid management.

It suppresses the expression of proteins related to cell proliferation and survival, impacting cyclins and anti-apoptotic factors. High doses of EGCG in rat ELT3 UF cells significantly inhibited cell growth, leading to a 40% decrease in proliferation and reduced the expressions of proliferation markers like *PCNA* and *CDK4* proteins. Pro-EGCG analogs have shown promise in inhibiting proteasome and *Akt* signaling pathways [75]. EGCG also inhibits the production and activity of pro-inflammatory molecules, mitigating the inflammatory response associated with UF growth. While there is limited and inconclusive evidence, some studies suggest that EGCG may play a role in reducing UF volume by inhibiting cell growth or inducing apoptosis in laboratory and animal models. However, translating these findings to human studies is in the early stages. A small pilot study showed reduced UF volume and symptom improvement in some participants with EGCG administration, but larger, well-designed clinical trials are needed to establish efficacy and optimal dosage [76]. The use of EGCG (800 mg/day) was suggested in our ESCAPE approach in order to manage UFs [10] (Figure 1).

The figure highlights the influence of dietary choices on uterine fibroid (UF) risk, emphasizing the importance of consuming fiber-rich vegetables such as leafy greens, cruciferous vegetables, carrots, sweet potatoes, and bell peppers, along with fruits like berries, citrus fruits, apples, pineapple, and kiwi. Furthermore, the inclusion of omega-3 fatty acids, specific fish, EGCG, vitamin D, and poultry products is underscored as an essential consideration of a UF-preventive diet.

### 3.7. Suboptimal Food Choices to Avoid

When it comes to nutrition and UFs, there are certain dietary factors that are believed to be less favorable and may potentially worsen the condition. While individual responses to specific foods may vary, it is generally recommended to limit or avoid certain foods. It is important to note that the impact of these dietary factors may vary among individuals, and it is best to consult with a healthcare professional or registered dietitian who can provide personalized recommendations based on specific needs and health conditions.

#### 3.7.1. Red Meat

Red meat contains high levels of saturated fat, which can increase inflammation and promote UF growth [66]. Red meat, particularly processed and unprocessed beef and pork, is known to contain high levels of saturated fats and potentially harmful compounds such as heterocyclic amines and polycyclic aromatic hydrocarbons, which can promote inflammation and hormonal imbalances in the body [77,78]. However, more research is needed to establish a definitive link between red meat consumption and UFs, as the existing evidence is limited and inconclusive. It is advisable for individuals to maintain a balanced and varied diet that includes a moderate intake of red meat while also emphasizing other nutrient-rich foods to support overall health and potentially minimize the risk of UFs.

#### 3.7.2. High-Fat Dairy Products

The role of high-fat dairy products in UFs is not yet well established, and more research is needed to understand their potential impact. However, it is believed that high-fat dairy products may have a negative influence on UFs due to their association with hormonal imbalances and inflammation [79]. Dairy products, particularly those high in fat, can contain high levels of estrogen and other hormones. These hormones can potentially affect the hormonal balance in the body, especially estrogen levels, which are believed to influence the development and growth of UFs [80]. High-fat dairy products can contribute to inflammation in the body, and chronic inflammation has been linked to the progression of various health conditions, including UFs [81].

While the evidence is limited, it may be beneficial for individuals with UFs to choose low-fat or skim dairy products as part of a balanced diet. This can help reduce the intake of hormones and saturated fats commonly found in high-fat dairy [82].

#### 3.7.3. Caffeine

The effect of caffeine on UFs is a topic that requires further investigation, as the available evidence is limited and conflicting. Caffeine is a stimulant that is commonly found in coffee, tea, energy drinks, and some sodas. Some studies suggest that high caffeine intake may be associated with an increased risk of developing UFs [83], while others have not found a significant association [66]. Caffeine is believed to affect estrogen levels in the body, which could potentially influence the growth and development of UFs [84]. It is worth noting that individual responses to caffeine can vary, and some women with fibroids may find that reducing their caffeine intake helps alleviate symptoms such as pain or HMB [85].

#### 3.7.4. Alcohol

The relationship between alcohol consumption and UFs is not fully understood and requires further research. While there is limited evidence on the direct effects of alcohol specifically on UFs, alcohol intake is known to have several general health implications [86]. Excessive alcohol consumption can lead to hormonal imbalances, including increased estrogen levels [87]. Moreover, alcohol is metabolized in the liver, and excessive alcohol consumption may impair liver function. The liver plays a crucial role in metabolizing hormones, including estrogen, and any disruption in its function could potentially impact hormone levels and contribute to UF development or progression [88,89,90]. It is important to note that moderate alcohol consumption, defined as up to one drink per day for women, may not have significant adverse effects on UFs or overall health. However, excessive or chronic alcohol intake can have detrimental effects on various aspects of health, including liver function and hormone balance. Women with UFs or those concerned about them should discuss alcohol consumption with their healthcare provider. They can provide personalized recommendations based on the specific health circumstances and help women make informed decisions about alcohol intake.

## 4. Sustainable Environmental Practices (Everyday Household Measures for Reducing EDC Exposure)

Emerging evidence indicates that EDCs present in various aspects of our daily lives can potentially have long-term effects on reproductive health [91]. It is crucial to adopt preventive measures to minimize environmental exposure to these harmful EDCs, based on scientific findings. By following simple guidelines, we can take proactive steps to protect ourselves from the potential risks associated with EDCs.

### 4.1. Indoor

Phthalates are a group of EDCs commonly used to enhance the flexibility and durability of plastics. While the potential link between exposure to these compounds and gynecological disorders requires further research to be fully understood, they have been identified as potential indicators of the risk of developing UFs [92]. Phthalates are available in consumer products, including food packaging, kitchen utensils, toys, PVC plastic products, flooring, outdoor furniture, raincoats, nail polish, facial washes, cosmetics, shower curtains, detergents, cleaning supplies, and even medical devices such as catheters and intravenous injection equipment [93]. Regulatory measures have been implemented by both the European Union and the United States to address the use of phthalates. The European Union, through regulations such as the Classification, Labelling, and Packaging (CLP) Regulation and REACH (Registration, Evaluation, Authorization, and Restriction of Chemicals), has imposed restrictions on phthalate applications [94]. Similarly, in the United States, several federal agencies such as the Consumer Product Safety Commission (CPSC) and the Food and Drug Administration (FDA) have enacted regulations. For instance, the use of phthalates exceeding 0.1% by weight in children’s toys and childcare products has been permanently prohibited due to their recognized toxicity [95].

Given the importance of minimizing exposure to phthalates, increasing research attention has been directed toward understanding the effects of phthalate exposure. Phthalates are rapidly metabolized through various pathways, including the kidneys/urine and the liver, and are typically eliminated from the body once exposure ceases. However, due to the current lack of a comprehensive implementation of resources, it is crucial to provide simple educational protocols and intervention strategies to promote future health and reduce health disparities [96]. Until better regulation is in place, making a few simple changes can have a significant impact on promoting health and minimizing phthalate levels. Women can be exposed to phthalates through various routes, including the ingestion of food or substances, absorption through the skin, and the inhalation of airborne dust containing phthalates [97,98]. Specific considerations must be taken for infants and daughters, such as the direct exposure of a developing fetus from a mother who has been exposed, ingestion through breast milk, and exposure during hospitalization through direct injection into the bloodstream via medical tubing or intravenous (IV) procedures [99].

By following these guidelines, women can minimize phthalate exposure and support the body’s natural ability to stay healthy such as by not using single-use water bottles, staying hydrated (drinking filtered water daily to ensure an adequate flushing of toxins from the body), including detox foods in the diet (certain foods, such as cruciferous vegetables (broccoli, kale, Brussels sprouts), asparagus, grapefruit, avocado, lemon, oranges, beets, and vitamin B-rich leafy greens), and supporting natural detoxification processes in the body [100]. Adding various antioxidant-rich foods, including cranberries, red grapes, turmeric (containing curcumin) and resveratrol, can help our bodies protect from cellular damage [101]), as well as adopting a plant-based diet (a large population study found that the frequent consumption of fast food is associated with higher levels of phthalates in urine). Choosing a plant-based diet can help reduce exposure to phthalates present in processed and packaged foods [102]; the diet helps with the intake of vitamins (compounds like vitamin C and E, quercetin, and NAC can be found in organic ones and can be helpful [103]) and fiber (fiber acts like a web that catches toxins in the gut and pulls them out for elimination). High-fiber foods like vegetables, fruit, legumes, whole grains, and seeds can improve regularity, trap toxins, and even balance hormones [104]. Removing shoes before entering the home, avoiding microwaving plastics, avoiding perfumed beauty and wellness products (phthalates are used to stabilize the fragrance in perfumes, enhancing its longevity [105]), avoiding washing plastics in the dishwasher, reducing beauty product use, avoiding nail polish (phthalates help prevent nail polish from peeling), reducing pesticide use, plugging holes under the sink or using trappers without plastics at home, washing hands regularly [106], and dusting and vacuuming often (phthalates can be inhaled from dust or fumes from any vinyl product, such as vinyl flooring and vinyl rugs, for example, in car seats and changing mats, also help with minimizing phthalate exposure. The production of gas and vapors by these products is called off-gassing. Vacuuming can be performed by repairing floors and windows in the home to decrease dust accumulation. Keeping the house clean, even with water flushing, can also help remove phthalates [107]; use High-Efficiency Particulate Air (HEPA) filter vacuums indoors. Watch what you eat, keep your food fresh (choose fresh whole foods over canned, frozen, and processed foods as much as possible [108]), and avoid air fresheners (prefer natural ventilation by opening windows or use homemade air fresheners). Women can create their own air freshener by adding a few drops of pure essential oil to a water bottle. Remove the top layer of food before usage (when purchasing food items like cheese or meat wrapped in plastic, this helps minimize potential exposure to phthalates) and limit your consumption of shellfish (the oceans are contaminated with microplastics, which can find their way into seafood, including mollusks like mussels and clams). Based on studies revealing high levels of microplastics in shellfish, reducing their consumption is advisable to minimize exposure [109]). Minimize the use of plastic food and beverage containers and reduce the consumption of high-fat dairy products (as phthalates have a higher affinity for fat and can be stored in adipose tissue). Opting for low-fat animal products can help reduce exposure to phthalates.

### 4.2. Minimizing Phthalate Exposure in Infants and Young Children

When it comes to children, it is important to pay attention to the following points, including choosing toys wisely (avoid purchasing polymer play dough, teethers, pacifiers, or toys that can fit entirely in a baby’s mouth; this is especially crucial for infants and children who are teething, as they tend to chew or suck on toys), look for brands that use alternative materials in the synthesis of their products (by being mindful of these practices, women can help minimize potential exposure to phthalates for both themselves and their children [110]), use recommended or pediatrician-approved baby care and avoid using baby cosmetics, limit exposure during pregnancy and breastfeeding (to protect the baby, it is essential to reduce the mother’s phthalate exposure, as phthalates can cross the placenta and be transferred to the fetus), avoid ready-packaged powdered milk in plastic cans, and opt for breastfeeding or choose powdered formula milk free from phthalates. Also, select phthalate-free lotions, creams, and powders [111] and take part in the Pregnancy Prevention Endocrine Disruptors (PREVENT) Interventional program. Programs like the Pregnancy Prevention Endocrine Disruptors (PREVENT) Interventional program focus on educating and supporting pregnant women to minimize exposure to harmful substances, including phthalates [112]. This report outlines practical solutions for shielding women and their babies from the dangers of phthalates. Given the significance of this issue, there is a strong recommendation to establish comprehensive guidelines for the benefit–risk assessment concerning phthalate exposure. These guidelines are especially crucial for individuals and healthcare providers.

### 4.3. Outdoor

It is highly recommended to consider the points below to diminish phthalate exposure, like by reading labels (always read product labels to check for high-molecular-weight phthalates). Look for terms like “phthalate-free” or “natural”. Avoid plastics labeled “non-stick” or “stain resistant” [33]. Plastics marked with recycling codes 1, 2, 4, or 5 are safer options, while those with codes 3, 6, and 7 should be used with caution [34]. Manufacturers may hide phthalates under the “fragrance” ingredient, making it advisable to check with them directly or their websites. As a guide, phthalates are usually identified on product labels by their more common names, including DEP (Diethyl Phthalate), DEHP (Di 2-ethylhexl Phthalate), DiNP (Diisononyl Phthalate), DIBP (Diisobutyl Phthalate), DiDP (Diisodecyl Phthalate), DnHP (Di-n-hexyl Phthalate), DnOP (Di-n-octyl Phthalate), DMP (Dimethyl Phthalate), DBP (Dibutyl Phthalate), BBzP (Benzyl butyl Phthalate), and BBP (Benzyl butyl Phthalate). Also, the safest solution is to Google specific products that do not contain phthalates [113,114]. Avoid getting stuck in traffic; otherwise, close the car windows, wear phthalate-free masks, reduce ambient phthalate levels in the environment to ensure cleaner air for women [115], and be cautious with the types of plastics and containers you use or purchase. Research shows that poultry, certain dairy products like ice cream and cheese (but not milk or yogurt), and fats often have higher phthalate concentrations than other foods [103]; filter the tap water, avoid heating food in plastic containers, as this can cause phthalates to leach into the food, and seek alternatives (opt for materials like glass, stainless steel, ceramic, or wood over plastic for storing food and drinks). Products labeled as free from specific EDCs, such as BPA, are preferred. Alternatively, consider plastics made from corn (PLA) or those made from polyethylene or polypropylene instead of vinyl or PVC [116,117]; utilize web-based and digital health interventions. Leverage online and digital health platforms to stay informed and reduce phthalate exposure and utilize educational tools (opt for phthalate-free products, and participate in support groups and meetings). It is important to establish guidelines for assessing the benefits and risks associated with phthalates. This includes monitoring the presence of phthalates and raising awareness among individuals and healthcare providers about their potential hazards [118].

## 5. Future Direction (under Investigation) of Natural Compound Usage

**Figure 2:**
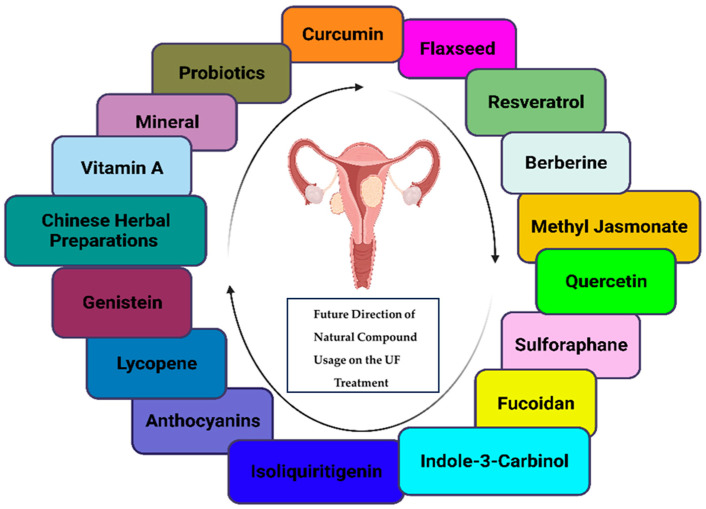
Natural compounds as a holistic approach for UF prevention and management. This figure highlights the potential of natural compounds in enhancing gynecological health and preventing UFs. Included in the list are curcumin, flaxseed, resveratrol, berberine, methyl jasmonate, quercetin, sulforaphane, fucoidans, indole-3-carbinol, isoliquiritigenin, anthocyanins, lycopene, genistein, Chinese herbal preparation, vitamin A, minerals, and probiotics, which contribute to a healthy gut microbiome and may consequently reduce the risk of UFs.

Natural compounds, synthesized by living organisms such as plants and animals, serve protective purposes and have potential therapeutic properties. Examples of natural compounds include alkaloids, terpenes, flavonoids, polyphenols, and fatty acids. By incorporating these compounds into one’s diet, individuals may potentially mitigate the risk of UFs and promote better gynecological health (Figure 2) [119,120].

### 5.1. Curcumin

*Curcumin* is a bright yellow chemical produced by plants of the *Curcuma longa* species. It is a natural phenol found in turmeric that has been studied for its various properties and potential benefits [121,122,123]. Curcumin exhibits anti-inflammatory, anticarcinogenic, and antioxidant properties [124]. It has been shown to inhibit cell proliferation, suppress fibrosis, and regulate apoptosis. These findings suggest that curcumin may have therapeutic potential in managing conditions such as UFs. However, further research is needed to fully understand its mechanisms of action and its effectiveness in this specific context [125]. Also, curcumin may potentially help alleviate symptoms associated with UFs due to its antioxidant activity, which helps protect cells from oxidative stress and damage [126]. While preliminary studies suggest a potential benefit of curcumin in UFs, more robust clinical trials are needed to determine its efficacy and optimal dosage [74]. It is important to consult with a healthcare professional before using curcumin or any other supplement for UFs to ensure it is safe and appropriate for the individual’s circumstances.

### 5.2. Flaxseed

Flaxseed (*Linaceae usitatissimum*) is rich in lignans, which are phytoestrogens known for their potential anti-inflammatory and antioxidant properties [127]. These properties have led to suggestions that flaxseed could offer benefits for UFs [128,129]. However, it is important to note that flaxseed may interact with certain medications or supplements. Additionally, due to its high fiber content, flaxseed should be consumed in moderation as excessive intake can cause digestive discomfort in some individuals [130,131]. As with any dietary intervention, it is advisable to consult with a healthcare professional before incorporating flaxseed into the routine [132].

### 5.3. Resveratrol

Resveratrol (RSV), (3,5,4′-trihydroxy-trans-stilbene), is a polyphenolic phytoalexin found abundantly in mulberries, peanuts, and grapes [133]. RSV has the ability to reduce the expressions of ECM-related proteins in primary human UFs. This finding highlights the potential of resveratrol as an anti-fibrotic therapy, suggesting its ability to mitigate fibrotic processes associated with UFs [134]. In addition, RSV exhibits inhibitory effects on human UF cell proliferation by targeting the crosstalk between integrin αvβ3 and IGF-1R [135]. This crosstalk inhibition mechanism suggests that RSV can interfere with the signaling pathways involved in UF cell growth, potentially offering therapeutic benefits in the management of UFs. A study revealed the inhibitory effects of RSV on the ELT-3-LUC xenograft model, highlighting its potential as an anti-fibrotic therapy for UFs. RSV reduced the expressions of ECM-related proteins in primary human leiomyoma cells, further supporting its potential therapeutic benefits for UFs [136]. RSV possesses pleiotropic activities, including anti-proliferative, pro-apoptotic, anti-carcinogenic, and antioxidant effects, which contribute to its potential efficacy in UF management [22]. These findings provide a foundation for future investigations aimed at unraveling the molecular mechanisms underlying the inhibitory action of RSV on UFs. Further studies are warranted to elucidate the precise mechanisms by which RSV exerts its inhibitory effects on UF and to explore its potential therapeutic applications.

### 5.4. Berberine

*Berberine* (BBR), which is found in such plants as *Berberis vulgaris* (barberry), is a natural alkaloid derived from plants, particularly *Scutellaria barbata*, and has a long history of use in traditional Chinese medicine [137]. BBR is known for its anti-inflammatory and anti-tumorigenic properties [138]. Notably, BBR has been found to selectively inhibit cellular proliferation and effectively block the proliferation induced by estrogen (E2) and progesterone (P4) in human UF cells, without causing cytotoxicity [139]. BBR significantly reduces the mRNA levels and proteins of *COX2*, *PTTG1*, *Ki-67*, *PCNA*, *Cyclin D1*, and *CDK1* in a dose-dependent manner. Importantly, these effects have been observed in HuLM cells while normal human uterine smooth muscle (UtSMC) cell lines were unaffected. This suggests that BBR holds promise as a targeted therapeutic option for managing UFs [140].

### 5.5. Methyl Jasmonate

*Methyl Jasmonate* (MJ), from jasmine oil derived from *Jasminum grandiflorum*, is a naturally occurring compound obtained from jasmine plants. It has been found to possess remarkable anti-*EZH2* (an enhancer of zeste homolog 2) activity when used in the treatment of UFs [141]. *EZH2* is an enzyme involved in gene regulation and has been implicated in UF development. The anti-*EZH2* activity of MJ suggests its potential as a therapeutic agent for UFs [142]. Further research is needed to explore the underlying mechanisms and evaluate the efficacy of MJ in the treatment of this condition [6]. In a study conducted by Ali et al., it was observed that MJ exhibited significant anti-proliferative effects on UFs cells, even at low concentrations, in comparison to myometrial cells. This suggests that MJ may hold promise as a potential therapeutic option for inhibiting the growth of UF cells specifically, while having a relatively lesser impact on normal myometrial cells [143]. Further research is required to explore the full potential of MJ and its mechanisms of action in UF treatment [144].

### 5.6. Quercetin

*Quercetin*, as a plant antioxidant flavonoid and flavanol source is derived from quercetum (oak forest). It is commonly found in various foods including onions, grapes, berries, cherries, broccoli, tea, lemon, tomato, onion leaves, strawberries, and citrus fruits [145]. Its presence in these natural sources highlights its wide availability in the diet and potential health benefits [146,147]. Quercetin contributes to the vibrant colors of fruits and vegetables, while its antioxidant properties make it valuable in promoting overall health and well-being [148]. Recent studies on quercetin, with anti-fibrotic activity effects on the *TGF-β/Akt/mTOR* signaling pathway and reduced *IL-6*, *VEGF* expression [149], have demonstrated the potential of quercetin in reducing collagen and fibronectin mRNA expressions in UFs. Moreover, quercetin has been found to modulate the migration pattern of UF cells, highlighting its potential as a beneficial compound in UF management [150,151].

### 5.7. Sulforaphane

*Sulforaphane*, an isothiocyanate, is abundant in various cruciferous vegetables such as broccoli, sprouting broccoli, Brussels sprouts, cabbage, cauliflower, collard greens, kale, kohlrabi, mustard, rutabaga, turnips, bok choy, and Chinese cabbage [152,153]. These nutritious plant foods serve as excellent natural sources of sulforaphane, showcasing its wide distribution in the diet. With its distinct health-promoting properties, sulforaphane contributes to the many potential benefits associated with consuming cruciferous vegetables [154]. *Sulforaphane* effectively reduced fibrosis by inhibiting the process of the epithelial–mesenchymal transition. This inhibition results in a significant decrease in the expression levels of *N-cadherin*, *vimentin* and *α-SMA* Moreover, sulforaphane has demonstrated its abilities to hinder myofibroblast formation induced by *TGF-β1* and to suppress the expression of integrins through both canonical and non-canonical *TGF-β* signaling pathways. Sulforaphane has exhibited its potential in reducing the expression of various cytokines, including *TNF-α* and *IL-6* [155,156].

### 5.8. Fucoidans

*Fucoidans*, sulfated polysaccharides derived primarily from *brown algae*, are a class of sulfated polysaccharides that can be found abundantly in different species of brown seaweeds and algae [157]. These natural compounds have been extensively studied for their remarkable properties, including antioxidant, anti-inflammatory, anti-angiogenic, and anti-cancer activities [158]. *Fucoidan* treatment results in a significant reduction in cell proliferation, as well as decreased collagen, fibronectin, vimentin, and *α-SMA* protein levels. The *β-catenin* translocation is inhibited by the natural chemical fucoidan, which significantly suppresses the *Smad2* and *ERK1/2* signaling pathways [159]. The unique composition of fucoidans and their potential therapeutic effects have garnered significant interest in the scientific community, paving the way for a further exploration of their diverse applications in health and wellness [160].

### 5.9. Indole-3-Carbinol

Broccoli, Brussels sprouts, cabbage, cauliflower, and kale are examples of cruciferous vegetables that naturally contain the natural chemical *indole-3-carbinol* (I3C). I3C, along with quercetin, exhibit the ability to regulate the expressions of ECM components, as well as the migration and proliferation of primary UF cells. Notably, a study found that I3C significantly reduces the mRNA expressions of collagen 1 and fibronectin, suggesting its potential as a therapeutic agent for UF management [150]. I3C affects several signaling pathways and target molecules, such as *PI3K/Akt/mTOR* and *NF-κB*, that control cell division, apoptosis, and angiogenesis in various recognized cancers, including UFs [161,162].

### 5.10. Isoliquiritigenin

*Isoliquiritigenin*, a phenolic compound found in various plant species, notably those within the licorice family such as *Glycyrrhiza uralensis*, *Mongolian glycyrrhiza*, and *Glycyrrhiza glabra*, as well as in common foods and alternative medicine products, has attracted attention for its potential therapeutic effects [163,164]. *Isoliquiritigenin* has demonstrated potential as a therapeutic drug for treating UF by efficiently inhibiting the proliferation of UF cells in a dose-dependent manner. This activity was linked to caspase-3 activation and the downregulation of Bcl-2 [165]. *Isoliquiritigenin* has a strong effect on macrophages’ inflammatory response; it suppresses the *TNF-α*-induced activation of adipocytes and inhibits *NF-κB* activation while stimulating peroxisome proliferator-activated receptor-γ [166].

### 5.11. Anthocyanins

*Anthocyanins*, which are vibrant water-soluble flavonoid pigments, contribute to the red, purple, blue, or black colors observed in various plants [167]. Fruits like blueberries, raspberries, and strawberries are particularly rich in these pigments [168]. The potent anthocyanins found in strawberries hold potential as a therapeutic or preventive option in the treatment of UFs [169]. However, to establish their effectiveness for women with UFs, additional animal studies and clinical trials are required [170].

### 5.12. Lycopene

Existing evidence indicates that women who consume a higher number of fruits and have a greater dietary intake of vitamin A may experience a decreased risk of developing UFs. These findings highlight the potential benefits of incorporating fruits and vitamin A-rich foods into one’s diet for the prevention of UFs. However, further research is needed to establish a definitive relationship and understand the underlying mechanisms involved [58]. *Lycopene* (from Neo-Latin *Lycopersicon*, the *tomato* species), a phytonutrient belonging to the carotenoid family, gives fruits and vegetables their characteristic orange and red color. It is abundantly found in commonly consumed foods such as tomatoes, carrots, papaya, and watermelon [171].

### 5.13. Chinese Herbal Preparations

Chinese medicine has long been utilized as a complementary or alternative approach to healthcare, with pharmaceutical industries recognizing the potential of plant-derived compounds for developing new drugs. Herbal preparations such as *Guizhi* fuling and *Nona roguy* have been traditionally used for the management of UFs [172]. In a study by Feng et al., the efficacy of *Rhizoma Curcumae* (RC) and *Rhizoma sparganii* (RS), both commonly used in traditional Chinese medicine, was demonstrated in preventing and treating UFs in rats, providing evidence of their therapeutic potential [173]. The combination of RC and RS demonstrated notable effects in reducing the expressions of collagen, fibroblast activating protein, and TGF-β, thereby suppressing cell proliferation. The combination treatment downregulated signaling factors, such as *AKT*, *ERK*, and *MEK*, involved in cellular proliferation pathways. In traditional Chinese medicine, this combination is known as *Gui Zhi Fu Ling*, or as the *Cinnamon* and *Poria* Formula, and has been used since the 3rd century AD in China to address various symptoms. The formula consists of five herbs: *Ramulus Cinnamomi*, *Poria*, *Semen Persicae*, *Cortex Moutan*, and *Peoniae rubrae radix or Peoniae alba radix* [172].

### 5.14. Genistein

*Genistein* was isolated in 1899 from a dyer’s broom; *Genista tinctoria* L. is an isoflavone derived from soybeans and fava beans that exhibits estrogenic properties and has been implicated in the protection against hormone-related cancers. In a study conducted by Castro et al., the researchers investigated the effects of genistein on tumor and normal UtSMC. The findings revealed the induction of novel cell death pathways by genistein, which could potentially be targeted to inhibit UF growth in vivo. These results shed light on the potential therapeutic implications of genistein in the management of UFs [174,175].

### 5.15. Vitamin A

Vitamin A has been shown to have beneficial effects on UFs by reducing cell proliferation, inhibiting ECM formation, and promoting apoptosis. Studies have demonstrated that vitamin A can effectively suppress UF cell growth and reduce the sizes of fibroids. It exerts its anti-proliferative effects by regulating cell cycle progression and inhibiting the expression of genes involved in UF development. Additionally, vitamin A promotes apoptosis, a process of programmed cell death, in fibroid cells, thereby promoting their elimination. These findings highlight the potential of vitamin A as a therapeutic approach for managing UFs [176,177].

### 5.16. Minerals

Selenium (Se), an essential trace element in the human diet, plays a crucial role in antioxidant function through the incorporation of selenocysteine residues into *ROS*-detoxifying enzymes such as glutathione peroxidases (*GPx*) and thioredoxin reductases [178]. By exerting its antioxidant properties, Se helps protect cells from oxidative damage. In avian studies, supplementation with Se has been shown to reduce the expression of heat shock protein 70 (*Hsp70*) in tissue. Notably, dietary supplementation with Se has been found to decrease the sizes of spontaneously occurring UFs in the oviduct of Japanese quail. These findings suggest that Se supplementation may have a potential therapeutic effect in reducing the size of leiomyoma in avian models [179,180].

Magnesium (Mg), an essential mineral, is involved in the proper functioning of muscles and nerves and has been implicated in the regulation of estrogen levels and inflammation reduction. Emerging research suggests a potential inverse relationship between Mg intake and the risk of developing UFs. Dietary sources rich in Mg include nuts, seeds, whole grains, and leafy green vegetables, and supplementation is also available. By incorporating Mg into one’s diet, individuals may potentially support their overall health and reduce the risk of UFs [181,182].

### 5.17. Probiotics

The gut microbiota consists of a complex community of microorganisms, including bacteria, fungi, and viruses, that coexist in symbiosis within the human gastrointestinal tract [183]. Probiotics, which are beneficial bacteria, can be acquired through various dietary sources like yogurt, kefir, fermented vegetables, or supplements, and contribute to maintaining a healthy gut microbiome [184]. Emerging research has proposed a potential association between imbalances in gut bacteria and the development of UFs. By promoting a balanced and diverse gut microbiota, incorporating probiotics into one’s diet may potentially support overall gut health and help mitigate the risk of UFs [185]. They should not be relied upon as a substitute for medical treatment or as the sole method of managing UFs. In addition, the consumption of whole grains such as brown rice, quinoa, and whole wheat bread can play a role in regulating estrogen levels in the body. By incorporating whole grains into the diet, women can help maintain a hormonal balance and potentially reduce the risk of UFs.

## 6. Conclusions

This paper highlights the pressing concern of UFs and introduces the innovative method termed LIFE UP awareness for their management. UFs are prevalent, disproportionately affecting women of color and significantly compromising their QOL. Conventional medical practices often entail invasive surgical procedures, leading to elevated recurrence rates and substantial healthcare expenditures.

The preventative strategy outlined in this review underscores evidence-based tactics for the early stage or mild management of UF pathology, offering a promising departure from the prevailing paradigm. Centered on prevention, LIFE-UP awareness offers a comprehensive framework with potential to forestall the development of UFs, enhance reproductive health, and diminish the necessity for invasive interventions. It promises substantial cost savings for both individual patients and healthcare systems.

Given UFs’ widespread prevalence, influence on fertility, potential complications, and ongoing research, acknowledging the significance of UFs is imperative. The identification of contributory risk factors represents a pivotal stride in the effective management of UFs, empowering healthcare practitioners and individuals to take preemptive measures to mitigate their impact. Strategies such as maintaining a healthy body mass index, following a balanced diet, regulating hormonal profiles, and regular medical checkups can mitigate the risk and severity of UFs.

In conclusion, adopting UF management, as advocated by “The LIFE-UP awareness” not only holds the promise of improved individual patient outcomes, but also charts a course for prospective scientific inquiries and policy initiatives in this critical domain of women’s health. Prioritizing prevention and early intervention can enhance the well-being of countless women affected by UFs and reduce the financial strain on healthcare systems.
